# Role of Vigilin and RACK1 in dengue virus-*Aedes aegypti-Wolbachia* interactions

**DOI:** 10.1128/msphere.00482-24

**Published:** 2024-12-23

**Authors:** Guijie Wang, Mazhar Hussain, Zhi Qi, Sassan Asgari

**Affiliations:** 1 Australian Infectious Disease Research Centre, School of Biological Sciences, The University of Queensland, Brisbane, Queensland, Australia; University of Michigan, Ann Arbor, Michigan, USA

**Keywords:** *Aedes aegypti*, mosquito, dengue virus, *Wolbachia*, Vigilin, RACK1

## Abstract

Vigilin is a large and evolutionary conserved RNA-binding protein (RBP), which can interact with RNA through its KH domain. Vigilin is, therefore, a multifunctional protein reported to be associated with RNA transport and metabolism, sterol metabolism, chromosome segregation, carcinogenesis, and heterochromatin-mediated gene silencing. The receptor for activated C kinase 1 (RACK1) is another highly conserved protein involved in many cellular pathways. Functional studies in human cells indicated that RACK1 interacts with Vigilin to promote dengue virus (DENV) replication. Both proteins are associated with the endoplasmic reticulum. Here, we investigated the significance of Vigilin and RACK1 homologs in *Aedes aegypti* mosquitoes concerning DENV replication and *Wolbachia* infection. We identified the homologs of the two genes in *Ae. aegypti* (*AeVigilin* and *AeRACK1*), which were upregulated in DENV-infected Aag2 cells and mosquitoes, and silencing them in Aag2 cells resulted in reduced DENV replication. Co-immunoprecipitation showed that AeRACK1 and AeVigilin interact in mosquito cells. Interestingly, we also found upregulation of both genes in a *Wolbachia*-infected cell line (Aag2.*w*AlbB). Furthermore, silencing *AeVigilin* and *AeRACK1* in Aag2.*w*AlbB cells reduced DENV replication but increased *Wolbachia* density. However, we did not find a significant effect on DENV replication after silencing both genes in *Ae. aegypti* mosquitoes. Overall, our results support the involvement and significance of AeVigilin and AeRACK1 in DENV replication in *Ae. aegypti*.

**IMPORTANCE:**

Dengue virus (DENV), transmitted mainly by *Aedes aegypti* mosquitoes, poses significant health risks. Identifying factors involved in the virus replication in mosquitoes and human hosts is essential for devising control measures. In this study, we show that Vigilin and the receptor for activated C kinase 1 (RACK1), two proteins shown to play a role in the replication of DENV in human cells, are induced in mosquitoes and cell lines following DENV replication. Both proteins reside in the cytoplasm, where they interact similarly to human cells. Silencing the genes in mosquito cells significantly reduced virus replication. Furthermore, we found that both genes are induced in mosquito cells transinfected with *Wolbachia*, a bacterium that blocks DENV replication. The results help better understand the role of the common factors supporting DENV replication in mosquitoes and human cells.

## INTRODUCTION

Mosquitoes are responsible for the most significant vector-borne diseases. Arthropod-borne viruses (arboviruses) such as Yellow fever virus, Chikungunya virus, Zika virus, and dengue virus (DENV), transmitted by *Aedes* mosquitoes, pose major threats to public health. Of the arboviruses mentioned, an estimated 50–100 million symptomatic DENV infections occur each year, posing the greatest burden to public health (1). DENVs comprise four serotypes based on the antigenicity of the viral envelope protein: DENV1 to DENV4 (2). Of these four serotypes, DENV-2 is known to cause the most severe cases of dengue hemorrhagic fever/shock syndrome, for which effective vaccines and targeted therapies are still lacking (3).

*Wolbachia pipientis* is a Gram-negative endosymbiotic bacterium that infects a wide range of invertebrates, including nematodes and arthropods (4, 5). *Wolbachia* inhibits the replication of a variety of RNA viruses, including DENV and other vector-borne viruses, in mosquitoes (6, 7); however, it is not naturally present in *Aedes aegypti* mosquitoes, the main vector of DENV (8). *Wolbachia* strains isolated from natural hosts such as *Drosophila melanogaster* and *Aedes albopictus* have been introduced into *Ae. aegypti* to reduce DENV transmission (9, 10). The virus blocking mechanism(s) appears to be variable depending on the combinations of the host species, *Wolbachia* strain, virus strain, and duration of the *Wolbachia*-host association, which include competition for resources (11–15) and space (16), induction of the immune and antiviral responses (17–19), or *Wolbachia* factors (20).

The endoplasmic reticulum (ER) is the largest organelle and plays a role in many cellular processes and during viral replication. Following entering the host cell through endocytosis or receptor-mediated entry, the DENV genome is released into the host cell’s cytoplasm and translated by ribosomes at the rough ER (2). To promote DENV replication, the virus induces dramatic rearrangements of the ER membrane (2, 21). *Wolbachia* can be found in various cells and tissues, including the central nervous system, relies on microtubules for intracellular transport, and is closely associated with the ER (22, 23). However, its tissue distribution varies depending on the *Wolbachia* strain and host species. Therefore, *Wolbachia* influences the ER system and may compete with DENV for resources and space in the ER to exert its virus blocking.

Vigilin is evolutionarily a highly conserved protein found in many eukaryotes, such as yeasts, nematodes, *Drosophila*, and vertebrates (24, 25). Vigilin, as a high-density lipoprotein-binding protein, is the largest RNA-binding protein (RBP) in the protein family with a KH domain, which interacts with RNA through this domain (26, 27). Although Vigilin is reported to exist mainly in the cytosol, bound to free ribosomes, a portion of Vigilin is detected in the ER fraction (28, 29). Vigilin has been reported to bind directly to rRNA and is preferentially enriched in binding to a subset of cellular mRNAs encoding secreted proteins, suggesting a potential role for Vigilin in translation (28). The receptor for activated C kinase 1 (RACK1) is a highly conserved protein with a core component of the 40S ribosomal subunit and contains seven WD40 domains that mediate protein-protein interactions (30). RACK1 is the best-characterized member of the WD multidomain scaffold protein family (31) and has been reported to interact with many cellular pathways, such as tyrosine kinase Src (32), receptor tyrosine kinase (33), and cAMP/protein kinase A (PKA) (34).

Reported studies have mainly focused on the functional role of Vigilin and RACK1 in virus-infected human cells, and there are no reports related to this in *Ae. aegypti*. Functional studies in human cells indicated that RACK1 interacts with the RNA-binding proteins Vigilin and SERBP1 to promote DENV replication (35). In this work, we investigated the effects of DENV and *Wolbachia* on the expression of *AeVigilin* and *AeRACK1* in *Ae. aegypti* and how their silencing affects *Wolbachia* density and replication of DENV *in vitro* and *in vivo*. The results provide further insight into the mosquito genes involved in DENV replication and *Wolbachia*-virus-mosquitoes interactions.

## MATERIALS AND METHODS

### Mosquitoes, cell cultures, and virus infection

*Aedes aegypti* mosquitoes were maintained at 28°C, a 12 h light/12 h dark cycle with relative humidity ranging between 65% and 70% on 10% sugar water *ad libitum*. Artificial feeding of female mosquitoes was carried out using glass feeders with blood obtained from the Australian Red Cross (UQ ethics HE000850) and equilibrated to 37°C. *w*AlbB-transinfected and tetracycline-cured *Ae. aegypti* mosquitoes (36) were used for analyzing *AeVigilin* and *AeRACK1* expressions and RNA was extracted at 2, 6, and 12 days post-emergence (dpe). In all our experiments, we used female mosquitoes.

Aag2 and Aag2.*w*AlbB cells were maintained at 27°C in a 1:1 mixture of Mitsuhashi–Maramorosch (HIMEDIA, cat# IML002) and Schneider’s Drosophila Medium (Invitrogen, cat# 21720024), supplemented with 10% fetal bovine serum (Interpath, cat# AUFBS/PG DIP) (37). Aag2 and Aag2.*w*AlbB cells were infected at 1 MOI using ET300 DENV-2 stock prepared by amplification in *Aedes albopictus* C6/36 cells, which were grown in RPMI-1640 medium (Invitrogen, cat# 11875093) supplemented with 5% FBS. A 12-well plate was prepared for infecting cells and mock-infected cells with the medium containing 2% FBS were used as control during the experiment.

### Gene homology analyses

Alignment of Vigilin and RACK1 amino acid sequences of several organisms was carried out using the MEGA 6.0 (http://www.megasoftware.net/) and GENDOC (http://www.nrbsc.org/downloads/gd322700.exe). The GenBank accession numbers of the sequences used in the alignment are AeVigilin (*Aedes aegypti*, XP_001659185.1), SpVigilin (*Schizosaccharomyces pombe*, CAA19118.2), ScVigilin (*Saccharomyces cerevisiae* S288C, DAA08719.1), CsVigilin (*Clonorchis sinensis*, KAG5453338.1), DmVigilin (*Drosophila melanogaster*, NP_477039.1), XtVigilin (*Xenopus tropicalis*, NP_001006854.1), CHICK-Vigilin (chicken, P81021.1), and human-Vigilin (*Homo sapiens*, NP_001307895.1). The GenBank accession numbers of the sequences used in the alignment are AeRACK1 (*Ae. aegypti*, XP_001663282.1), SpRACK1 (*S. pombe*, CAB11079.1), ScRACK1 (*S. cerevisiae*, DAA10013.1), DmRACK1 (*D. melanogaster*, AAB72148.1), XtRACK1 (*X. tropicalis*, NP_001004946.1), and human-RACK1 (*H. sapiens*, NP_006089.1).

### RNA extraction and reverse transcription quantitative PCR

Total RNA was extracted from mosquitoes and cells using the Qiazol reagent according to the manufacturer’s instructions (Qiagen, cat# 79306). Extracted RNA was treated with TURBO DNase (Invitrogen, ThermoFisher Scientific, cat# AM2238). cDNA was synthesized with an M-MuLV reverse transcriptase kit (New England BioLabs, cat# M0253L) using specific primers for DENV-2 and oligo-dT primers according to the manufacturer’s instructions. A two-step quantitative PCR (qPCR) was performed with two technical and at least three biological replicates using QuantiFast SYBR Green PCR Kit (Qiagen, cat# 204056) in a Qiagen Rotor-Gene Q under the following conditions: 95°C for 30 s, and 40 cycles of 95°C for 10 s and 60°C for 45 s, followed by the melting curve (68°C to 95°C). The ribosomal protein S17 (RPS17) was used for normalizing the qPCR data. The primer sequences to the gene are shown in Table 1.

**TABLE 1:** Primers used in this study

Primer name	Sequence (5′–3′)
*w*AlbB-*wsp*-qF	ATCTTTTATGGCTGGTGGTGCT
*w*AlbB-*wsp*-qR	GGAGTGATAGGCATATCTTCAAT
DENV2-qF	GGTATGGTGGGCGCTACTA
DENV2-qR	CAAGGCTAACGCATCAGTCA
*AeRPS17*-qF	CACTCCGAGGTCCGTGGTAT
*AeRPS17*-qR	GGACACTTCGGGCACGTAGT
*GFP*-qF	CCCAAGCTTCGCCACCATGGTGAGCAA
*GFP*-qR	CGGGGTACCCTTGTACAGCTCGTCCATGC
*AeVigilin*-qF	TCTGTTCGCACGACTACG
*AeVigilin*-qR	AAGAAGCATCCACCTGTT
*AeVigilin*-RNAiF	TAATACGACTCACTATAGGGAGAATAGCGAAACTGGAAAAC
*AeVigilin*-RNAiR	TAATACGACTCACTATAGGGAGATAGTACTTGGGTGGGATC
*AeRACK1*-qF	CTCGTCGGACGGTAACTACG
*AeRACK1*-qR	ATTTGACGGTTGTCGACGGA
*AeRACK1*-RNAiF	TAATACGACTCACTATAGGGAGAGATCCCGTGACAAGACCATCA
*AeRACK1*-RNAiR	TAATACGACTCACTATAGGGAGAGTTTGGCGAGAAGCACAAGG

Reverse transcription quantitative PCR (RT-qPCR) data were analyzed using the relative expression ratio method [ratio = (*E*_target_)^ΔCP^_target(control – sample)_/(*E*_ref_)^ΔCP^_ref(control – sample)_], as described previously (38). To calculate fold changes, the ΔΔCP value was determined by normalizing the ΔCP value of each experimental sample to the ΔCP value of the control sample as above. The control sample was set as the calibrator, with its expression level adjusted to 1, and the transcript levels in the treatments were expressed as fold changes relative to the control.

### Silencing *AeVigilin* and *AeRACK1* and *Wolbachia* density determination

The potential effect of AeVigilin and AeRACK1 on DENV-2 replication in Aag2 cells and *Wolbachia* density in Aag2.*w*AlbB cells was analyzed through RNA interference (RNAi)-mediated gene silencing of the genes. First, to produce *in vitro* dsRNAs, primers with T7 promoter sequences were designed to CDS of *AeVigilin* and *AeRACK1* for amplifying PCR fragments (450 bp). PCR primers are listed in Table 1. PCR products, after confirmation by sequencing, were used to generate dsRNAs using a MEGAscript T7 transcription kit (ThermoFisher Scientific, cat# AM1333). dsRNA to the *GFP* gene was used as a control. Transfection of Aag2 cells was performed using Cellfectin II (Invitrogen, cat# 10362100) and serum-free transfection medium. Transfection was performed in 12-well plates in replicates, including Cellfectin II, 2 μg of dsRNAs of *GFP*, *AeVigilin*, and *AeRACK1*. Cells were cultured at 27°C and infected with DENV at MOI of 1 after double transfections (2 days between the second transfection and viral infection). Supernatants and cells were collected after 3 days post-infection (dpi).

*AeVigilin* and *AeRACK1* were also silenced in Aag2.*w*AlbB cells to assess the effect on *Wolbachia* density by using the same dsRNA transfection procedure mentioned above. For *Wolbachia* density, genomic DNA was first extracted from the transfected cells using Econospin columns (Epoch Life Sciences, cat# 1920-250), following a protocol described previously (39). Two-step qPCR and melting curve analysis of Aag2 and Aag2.*w*AlbB DNA was performed using previously developed primers targeting the *AeRPS17* gene and the *Wolbachia surface protein gene* (*wsp*) as above (Table 1) (40).

### Mosquito injection and DENV infection

One-day-old female mosquitoes were placed on ice for 5 min to anesthetize and injected with 1 µg dsRNA of *GFP*, *AeVigilin,* or *AeRACK1* into the thorax by using a Nanojet III (Drummond) with a pulled glass needle. Two days after injection, mosquitoes were fed human blood donated by the Red Cross spiked with 10^7^/mL DENV-2 using glass feeders. Blood-fed mosquitoes were collected and maintained at 27°C with about 75% humidity on a 10% sugar solution for four days. RNA was extracted from individual mosquitoes and analyzed with RT-qPCR as described above.

### Focus forming assay for DENV-2 virion titration

To titrate DENV virions focus forming assay was used as described previously (41). Briefly, C6/36 cells were seeded in 96-well plates and infected with serial dilutions of supernatants (10 µL/well) collected from triplicate experiments. For titration of virions in mosquitoes, the body of each mosquito was homogenized in 500 µL of grinding medium (RPMI 1640, supplemented with 2% FBS, 1% Pen-Strep, 1% Fungizone), followed by centrifugation at 7,600 × *g* for 5 min, at 4°C. The supernatant (100 µL/well) was used to inoculate C6/36. Plates were first incubated at room temperature on a rocker for 1 h and then incubated at 37°C for an extra hour. At 3 dpi, cells were fixed in ice-cold 80% acetone in PBS for 20 min at −20°C and then dried overnight. The following day, cells were blocked with 5% skim milk in PBST (phosphate-buffered saline with 0.1% Tween 20) at 37°C for 30 min. This was followed by incubating cells with a monoclonal antibody specific to DENV-2 E protein, 4E11 (1:1,000) in 0.1% skim milk in PBST for 2 h at 37°C. After that, plates were washed three times with PBST followed by probing with secondary antibody IRDYE 800CW Goat anti-Human (1:2,500) (LI-COR) for 1 h at 37°C. Plates were washed three times with PBST and dried as above and scanned for foci detection and counting by LI-COR Biosciences Odyssey Infrared Imaging System.

### Isolation of cytoplasmic and nuclear fractions

The nuclear and cytoplasmic fractions were isolated using a PARIS kit (Invitrogen, cat# AM1921) according to the manufacturer’s instructions. Briefly, cells were washed with PBS and pelleted at low speed. They were then gently resuspended in 300 µL of ice-cold cell fractionation buffer, incubated on ice for 10 min, and centrifuged at 350 × *g* at 4°C for 5 min. The pellet contained the nuclear fraction, which was resuspended in 300 µL cell disruption buffer, vortexed vigorously, and used as the nuclear protein sample after adding 4× SDS-PAGE loading buffer. The supernatant contained the cytoplasmic fraction, which was mixed with 4× SDS-PAGE loading buffer.

### Western blot assay

Total proteins isolated from cells, or cytoplasmic and nuclear fractions, were subjected to western blot analyses. Protein samples were separated on 8% SDS-PAGE gels and transferred to nitrocellulose membranes. The membrane was blocked in Tris-buffered saline with Tween 20 (TBST; 10 mM Tris-HCl, pH 8.0, 150 mM NaCl, 0.05% Tween20) containing 5% non-fat dry milk, followed by three washes in TBST, and then incubated in TBST-1% non-fat dry milk with the primary antibody for 2 h, washed three times in TBST, and then in TBST-1% non-fat dry milk with the secondary antibody for 1 h. The polyclonal antibody to *Drosophila melanogaster* Vigilin was a kind gift of Dr. Fernando Azorin (42) and was used at 1:5,000 dilution. Polyclonal anti-RACK1 (ThermoFisher Scientific, cat# PA517484), anti-hnRNP (heterogeneous nuclear ribonucleoprotein) (Abcam cat# GR150878-1), and anti-rabbit antibody conjugated with alkaline phosphatase (Sigma, cat# A3937) were all used at 1:5,000 dilutions, respectively. The blots were developed by 1-Step NBT/BCIP substrate solution (ThermoFisher Scientific, cat# 34042) according to the manufacturer’s instructions.

The density of bands for Vigilin, RACK1, and Tubulin were measured using ImageJ, and ratios of Vigilin/Tubulin and RACK1/Tubulin were calculated. The results were plotted using GraphPad Prism.

### Co-immunoprecipitation

To determine if AeRACK1 interacts with AeVigilin we used co-immunoprecipitation using Aag2.*w*AlbB cells in which both proteins are upregulated (see Results). For this, cells were lysed in 1× RIPA cell lysis buffer (ThermoFisher Scientific) and the lysate was incubated with the anti-RACK1 or anti-tubulin (as a negative control: Sigma cat# T3559) antibody overnight at 4°C with gentle shaking. Protein A beads (50 µL) were added to the antibody-antigen lysates and incubated on ice for 1 h. Beads were collected by centrifugation at 10,000 × *g* for 1 min at 4°C followed by four washes with IP wash buffer (NaCl 150 mM, EDTA 1 mM, Triton X-100 1%, and Tris [pH 7.4] 10 mM). The captured proteins were eluted with 1× RIPA buffer, run on an 8% SDS-PAGE protein gel, and subjected to western blot hybridization with anti-Vigilin as above.

### Statistical analyses

GraphPad Prism 10 was used for data analysis and production of graphs. One-way analysis of variance (ANOVA) with Tukey’s post hoc comparisons test analysis or unpaired *t*-tests were used for statistical comparisons between treatments. All RT-qPCR results are representative of at least three independent experiments, each with three technical replicates.

## RESULTS

### Induction of *AeVigilin* and *AeRACK1* expression in DENV-infected cells and mosquitoes

**Fig 1:**
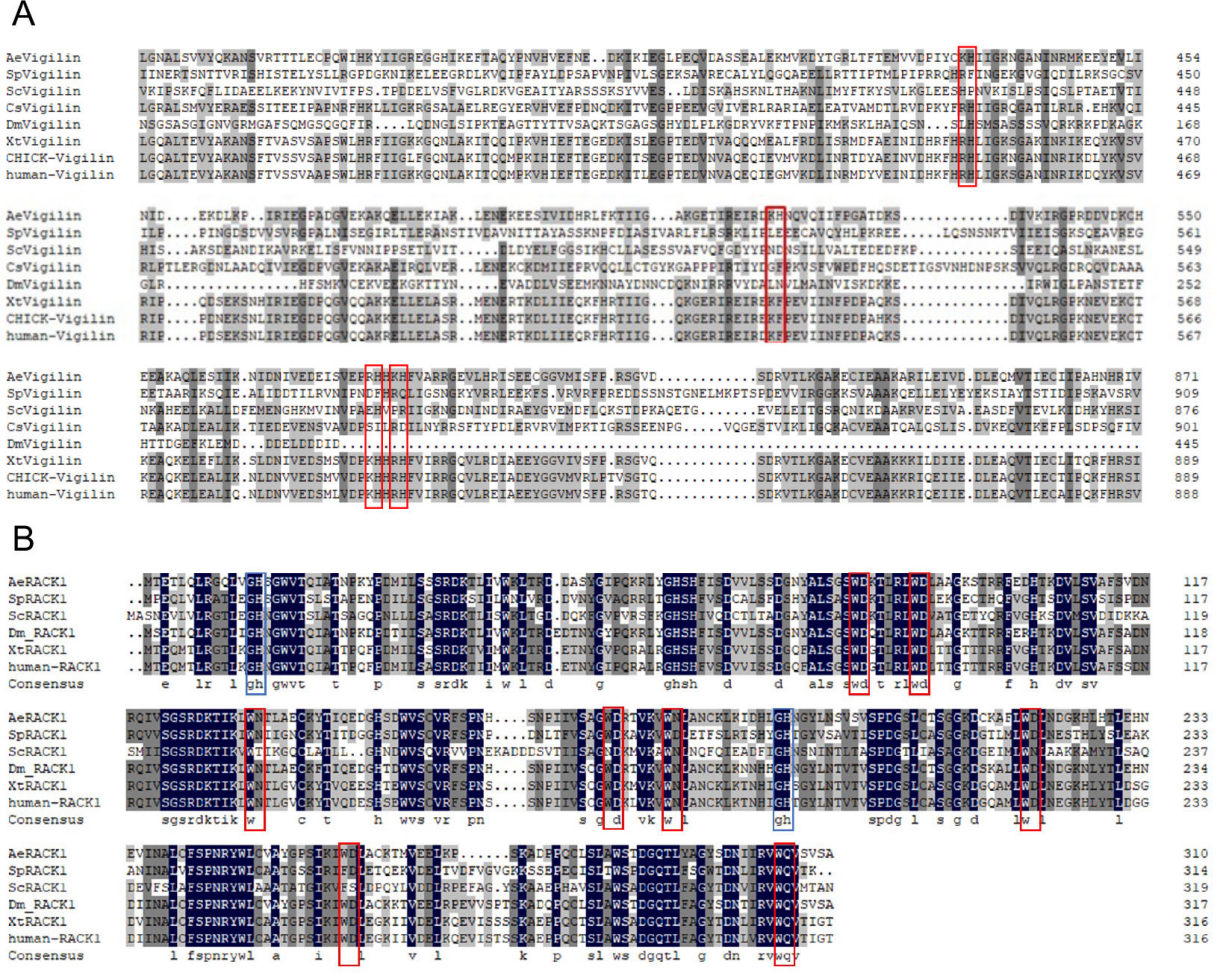
Multiple alignments of amino acid sequences of AeVigilin and AeRACK1. Homologous sequences to *Aedes aegypti* vigilin and RACK1 were downloaded from NCBI and used for multiple alignments, as described in Materials and Methods. Only the parts with domains are shown. (**A**) Vigilin and (**B**) RACK1 multiple alignments. AeVigilin contains four conserved KH canonical elements similar to human Vigilin (red boxed). Multiple amino acid sequence alignment also shows that AeRACK1 contains two conserved Gly-His (GH, blue boxed) and eight WD canonical elements similar to human RACK1 (red boxed). Ae, *Aedes aegypti*; Sp, *Schizosaccharomyces pombe*; Sc, *Saccharomyces cerevisiae*; Cs, *Clonorchis sinensis*; Dm, *Drosophila melanogaster*; Xt, *Xenopus tropicalis*; Chick, chicken. The accession numbers can be found in Materials and Methods.

*AeVigilin* and *AeRACK1* mRNA sequences and full-length proteins were obtained from the *Ae. aegypti* genome database VectorBase using BLAST search. The amino acid sequences of AeVigilin and RACK1 have an overall 44.70% and 76.90% identity to their homologs in yeasts, insects, and mammals (Fig. 1). AeVigilin is a protein composed of 1,246 amino acids with a molecular weight of 139.76 kDa and an isoelectric point of 6.18. Multiple amino acid sequence alignment results show that AeVigilin contains four conserved KH (or its equivalent) canonical elements similar to human-Vigilin. AeRACK1 is predicted as a protein of 311 amino acids with a molecular weight of 34.89 kDa and pI 7.86. Multiple amino acid sequence alignment shows that AeRACK1 contains two conserved Gly-His (GH) and eight WD (or its equivalent) canonical elements similar to human RACK1 (43).

**Fig 2:**
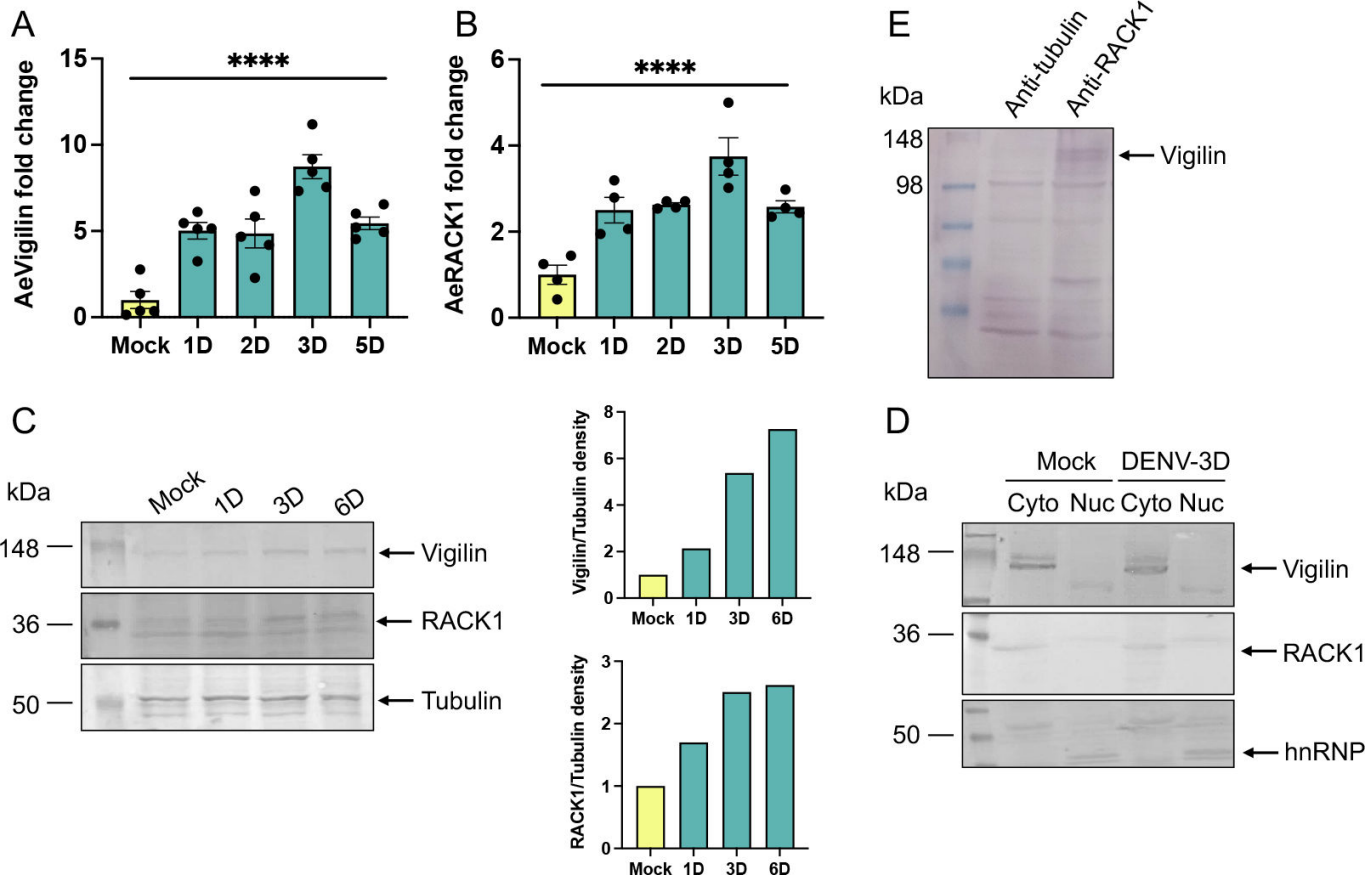
Expression of *AeVigilin* and *AeRACK1* following DENV infection in Aag2 cells. RT-qPCR analysis of the expression of (**A**) *AeVigilin* and (**B**) *AeRACK1* at 1 to 5 dpi with 1 MOI DENV. Noninfected cells (Mock) were collected and used as negative controls. Mock, 1 day post-mock infection. One-way ANOVA with Tukey’s post hoc comparisons test was used for statistical analysis. The error bars represent the standard error of the mean (SEM) of biological replicates. Asterisks indicate significant differences between compared samples. ****, *P* < 0.0001. (**C**) Protein samples from the same experiment (shown in panel A) were analyzed on a western blot using antibodies to Vigilin and RACK1, and tubulin as control. The blot is a representative of two independent experiments. The graphs show ratios of Vigilin and RACK1 band densities relative to tubulin measured by ImageJ. (**D**) Western blot analysis of the localization of AeVigilin and AeRACK1 in the cytoplasmic and nuclear fractions of Mock and DENV-infected (3 dpi) cells, respectively. The antibody to hnRNP was used as a control to confirm the nuclear fractions. Cyto, cytoplasmic; Nuc, nuclear. (**E**) Western blot analysis of co-immunoprecipitated proteins using anti-RACK1 as a trap and anti-tubulin as a control followed by detection with anti-Vigilin antibodies.

To explore *AeVigilin* and *AeRACK1* expression after DENV infection, Aag2 cells were infected with DENV at MOI of 1 and collected at different time intervals ranging from 1 to 5 dpi. Mock-infected cells were collected 1 day after mock infection. RT-qPCR showed no significant change in the expression levels of the genes between 1, 3, or 5 days post-mock infection (data not shown). RT-qPCR analysis of RNA collected from cells showed significant upregulation of both *AeVigilin* and *AeRACK1* transcript levels with prolonged infection (Fig. 2A and B). The expression of *AeVigilin* and *AeRACK1* at 3 dpi was significantly higher (one-way ANOVA, *P* < 0.0001 for both) compared with that observed in mock-infected cells, suggesting that *AeVigilin* and *AeRACK1* might be involved in DENV replication in Aag2 cells.

Expression of AeVigilin and AeRACK1 in DENV-infected cells was also examined at the protein level using western blot hybridization using protein-specific antisera. As expected, AeVigilin and AeRACK1 protein expression steadily increased following infection. The expression of AeVigilin and AeRACK1 at 3 dpi was higher compared with that observed in mock-infected cells (Fig. 2C). The results showed that DENV infection led to upregulation of AeVigilin and AeRACK1 at the protein level as well.

We wanted to explore whether DENV infection affects the localization of AeVigilin and AeRACK1 proteins in cells. The cytoplasmic and nuclear fractions of different treatments were separated using a kit. Western blot analysis showed that both proteins were only present in the cytoplasm in either mock or infected cells (Fig. 2D). The antibody to hnRNP was used as a marker for nuclear proteins. These results showed that AeVigilin and AeRACK1 are localized in the cytoplasm with or without DENV infection.

Previous research in human cells demonstrated the interaction of RACK1 with Vigilin (35). Using anti-RACK1 as a trap and anti-tubulin as a control, we utilized co-immunoprecipitation to determine if the two proteins interact in mosquito cells. Western blot analysis of the co-immunoprecipitated proteins using anti-Vigilin antibodies showed the presence of Vigilin only with anti-RACK1 co-immunoprecipitation and not with that of the control (Fig. 2E). This observation suggests that, like human cells, the two proteins interact in mosquito cells.

**Fig 3:**
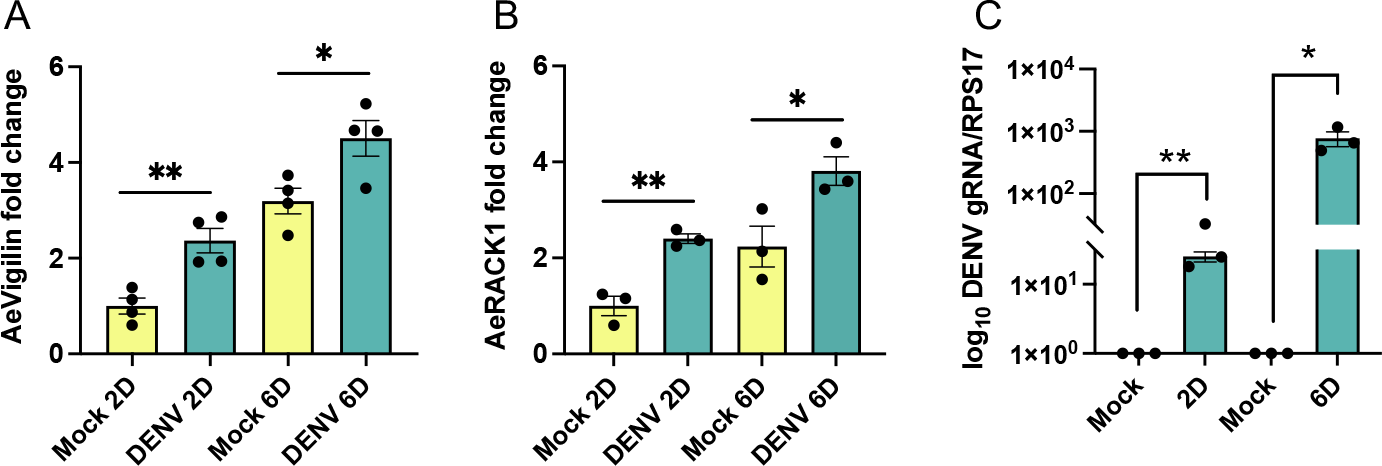
Expression of *AeVigilin* and *AeRACK1* following DENV infection in *Ae. aegypti* mosquitoes. RT-qPCR analysis of the expression of (**A**) *AeVigilin* and (**B**) *AeRACK1* at 2 and 6 dpi following DENV infection of mosquitoes, respectively. Mock 2D was used as the calibrator to calculate fold changes. (**C**) RT-qPCR analysis of the genomic RNA levels of DENV at 2 and 6 dpi following DENV infection of mosquitoes. The *t*-test was used for statistical analysis. The error bars represent the standard error of the mean (SEM) of biological replicates. Asterisks indicate significant differences between compared samples. *, *P* < 0.05; **, *P* < 0.01.

To explore whether viral infection also affects the expression of *AeVigilin* and *AeRACK1* in mosquitoes, we infected mosquitoes with DENV and collected them 2 and 6 dpi. RT-qPCR analysis of mosquitoes successfully infected with DENV showed that the transcript levels of *AeVigilin* and *AeRACK1* increased with increasing infection time (Fig. 3A and B). Compared with the control group, the expression of *AeVigilin* and *AeRACK1* increased significantly on 2 and 6 dpi after virus infection (*t*-test; AeVigilin 2 and 6 dpi *P* = 0.0041 and *P* = 0.0286, respectively. AeRACK1 2 and 6 dpi *P* = 0.0335 and *P* = 0.0393, respectively). DENV infection was confirmed in the mosquitoes (Fig. 3C). The results above suggest that DENV promotes the expression of *AeVigilin* and *AeRACK1* in cell lines and mosquitoes.

### Silencing *AeVigilin* and *AeRACK1* led to reduced DENV replication in Aag2 cells

**Fig 4:**
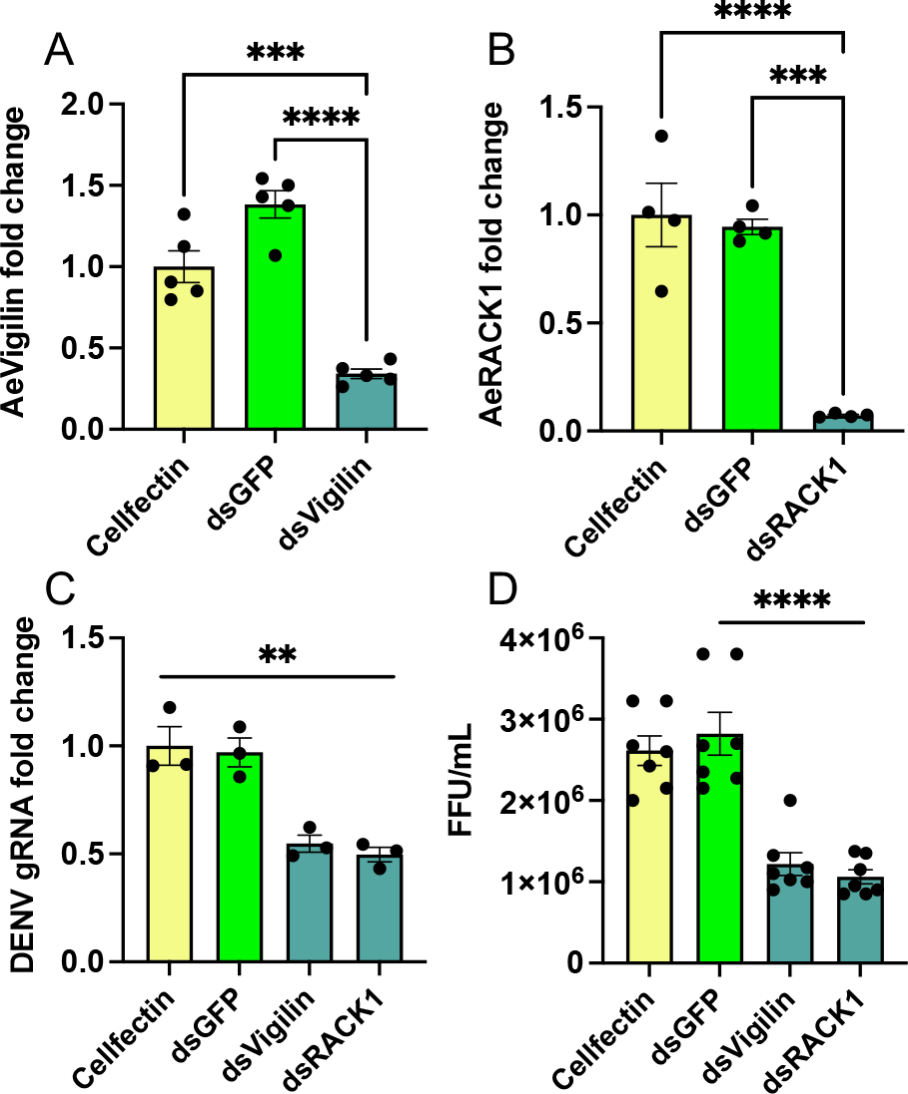
Silencing *AeVigilin* and *AeRACK1* by RNAi led to reduced DENV replication in Aag2 cells. RT-qPCR analysis of RNA collected from Aag2 cells transfected with dsRNAs of (**A**) *AeVigilin* and (**B**) *AeRACK1*, respectively. Cellfectin and *dsGFP* were used as controls. (**C**) The effect of *AeVigilin* and *AeRACK1* silencing on DENV gRNA levels was assessed by RT-qPCR analysis of RNA extracted from Aag2 cells treated as in panels **A and B** and infected with DENV at MOI of 1 for 3 days. (**D**) Focus-forming assays were performed using C6/C36 cells with DENV virions present in the supernatants collected from the experiment in panel **C**. One-way ANOVA with Tukey’s post hoc comparisons test was used to determine statistical significance. The error bars represent the standard error of the mean (SEM) of biological replicates. Asterisks indicate significant differences between compared samples. **, *P* < 0.01; ***, *P* < 0.001; ****, *P* < 0.0001.

To explore whether AeVigilin and AeRACK1 affect DENV replication, synthesized dsRNAs of *AeVigilin* and *AeRACK1* were used to transfect Aag2 cells, while dsRNA of *GFP* was used as control. First, we confirmed the silencing of *AeVigilin* and *AeRACK1* with RT-qPCR analysis showing a significant reduction of the target genes; *AeVigilin* (one-way ANOVA, *P* < 0.0001; Fig. 4A) and *AeRACK1* (one-way ANOVA, *P* < 0.0001; Fig. 4B). Next, using RNA from the same samples, DENV genomic RNA replication was assessed. RT-qPCR analysis showed that DENV replication was significantly reduced when *AeVigilin* and *AeRACK1* were silenced (one-way ANOVA, *P* = 0.0037 and *P* = 0.003, respectively; Fig. 4C). Supernatants collected from the experiment were subjected to focus forming assay. Consistent with the DENV genomic RNA (gRNA) results, DENV virus titers were significantly reduced in silenced cells (one-way ANOVA, *P* = 0.0005 and *P* < 0.0001, respectively) (Fig. 4D). Overall, the results suggest that AeVigilin and AeRACK1 are involved in DENV replication in Aag2 cells.

### *AeVigilin* and *AeRACK1* are upregulated in the *Wolbachia*-infected cell line but downregulated in *Wolbachia*-infected mosquitoes

**Fig 5:**
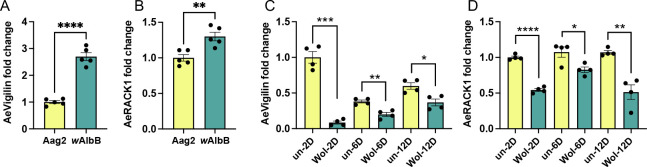
*Wolbachia* influences the expression of *AeVigilin* and *AeRACK1*. Analysis of the expression of (**A**) *AeVigilin* and (**B**) *AeRACK1* using RNA collected from Aag2 cells and Aag2.*w*AlbB cells (wAlbB). Comparison of the expression of (**C**) *AeVigilin* and (**D**) *AeRACK1* between uninfected (un) and *w*AlbB-infected (Wol) *Ae. aegypti* mosquitoes at 2, 6, and 12 dpe assessed by RT-qPCR. un-2D was used as the calibrator to calculate fold changes. The *t*-test was carried out to determine statistical significance between treatments. The error bars represent the standard error of the mean (SEM) of biological replicates. Asterisks indicate significant differences between compared samples. *, *P* < 0.05; **, *P* < 0.01; ***, *P* < 0.001; ****, *P* < 0.0001.

Results from the experiments described above suggested that *AeVigilin* and *AeRACK1* are likely pro-viral genes. We were interested in finding out the expression levels of these two genes in a cell line and mosquitoes infected with the *Wolbachia w*AlbB strain. RT-qPCR analysis of RNA extracted from Aag2 and Aag2.*w*AlbB cell lines suggested significantly higher expression of both genes in Aag2.*w*AlbB cells compared to Aag2 cells (*t*-test, *P* < 0.0001 and *P* = 0.0041, respectively; Fig. 5A and B). However, we found the expression of both genes in the *w*AlbB-infected mosquitoes was significantly reduced on days 2 (*t*-test, *P* < 0.0001 for both genes), 6 (*t*-test, AeVigilin *P* = 0.0022 and AeRACK1 *P* = 0.0237), and 12 (*t*-test, AeVigilin *P* = 0.0129 and AeRACK1 *P* = 0.0019) compared to uninfected mosquitoes (Fig. 5C and D). These results in mosquitoes are somewhat contradictory to those seen in the cell lines (Fig. 5A and B) and suggest that changes in *AeVigilin* and *AeRACK1* expression could be age/tissue/cell-specific. Aag2 and Aag2.*w*AlbB cells are rather homogenous cells, whereas mosquitoes are comprised of different cell types and tissues that may have differential expression levels of *AeVigilin* and *AeRACK1*. Our preliminary conclusion from the above results is that at the level of the cell line, *Wolbachia* can stimulate the expression of these two genes, while in mosquitoes, *Wolbachia* infection results in their downregulation.

### Silencing *AeVigilin* and *AeRACK1* affects DENV as well as *Wolbachia* density in Aag2.*w*AlbB cells

**Fig 6:**
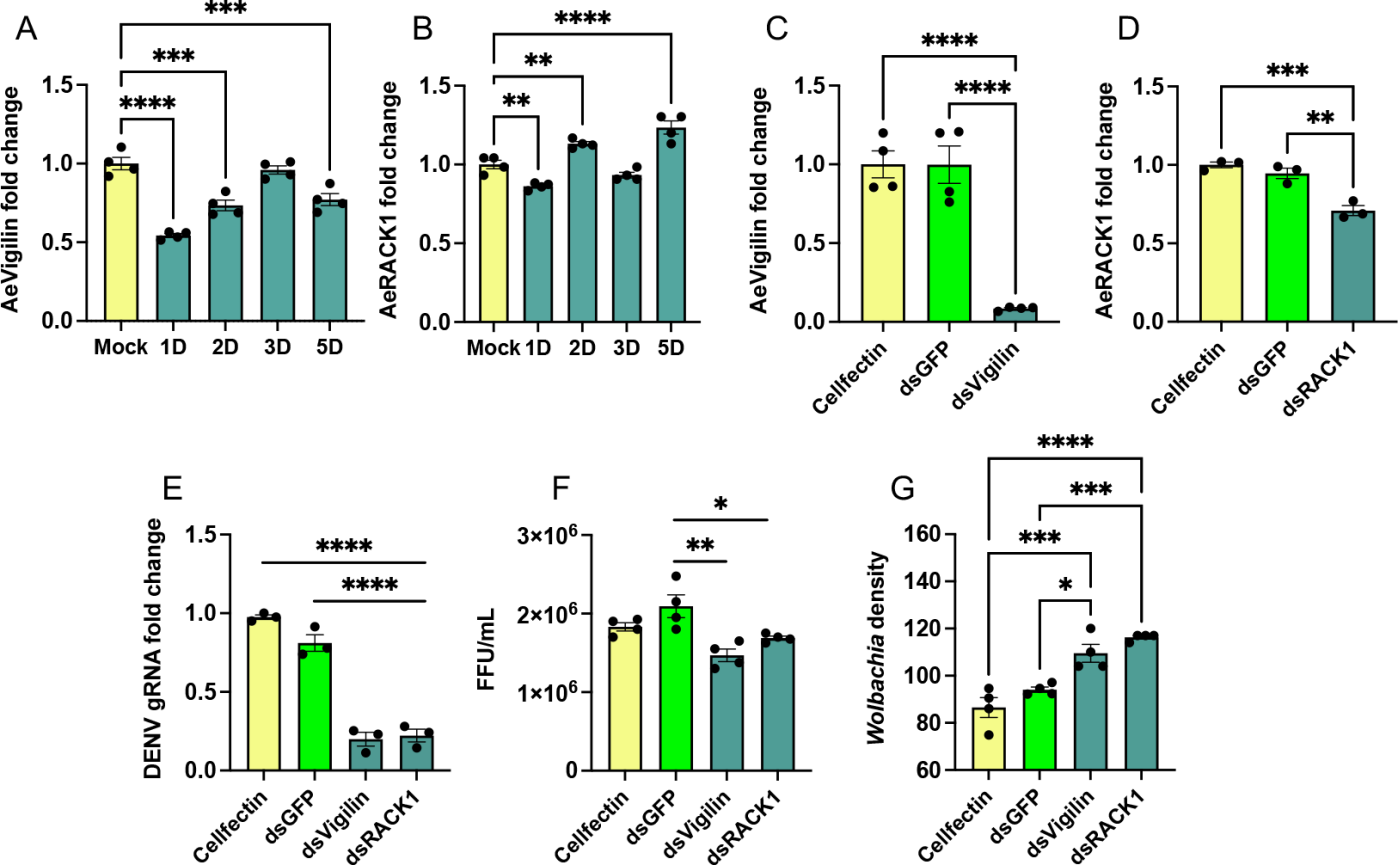
The effect of silencing *AeVigilin* and *AeRACK1* on DENV replication and *Wolbachia* density in *w*AlbB cells. RT-qPCR analysis of the expression of (**A**) *AeVigilin* and (**B**) *AeRACK1* at 1 to 5 dpi following DENV infection of *w*AlbB cells. RT-qPCR analysis of RNA collected from *w*AlbB cells transfected with (**C**) dsVigilin and (**D**) dsRACK1, respectively. Cellfectin and dsGFP were used as controls. (**E**) The effect of *AeVigilin* and *AeRACK1* silencing on DENV gRNA levels was assessed by RT-qPCR analysis of RNA extracted from Aag2.*w*AlbB cells treated as in panels **C and D** and infected with DENV at MOI of 1 for 3 days. (**F**) Focus-forming assays were performed using C6/C36 cells with DENV virions in the supernatants collected from the experiment in panel **E**. (**G**) qPCR analysis of DNA extracted from dsVigilin and dsRACK1 transfected and DENV-infected Aag2.*w*AlbB cells. A one-way ANOVA test with Tukey’s post hoc comparisons test was carried out to determine statistical significance among groups. The error bars represent the standard error of the mean (SEM) of biological replicates. Asterisks indicate significant differences between compared samples. *, *P* < 0.05; **, *P* < 0.01; ***, *P* < 0.001; ****, *P* < 0.0001.

Since we observed upregulation of *AeVigilin* and *AeRACK1* in DENV-infected *Wolbachia*-free Aag2 cells, we analyzed their expression in DENV-infected Aag2.*w*AlbB cells to find out if *Wolbachia* affects the expression of the genes during DENV infection. Interestingly, there was significant downregulation of *AeVigilin* in the cells compared to mock-infected cells at 1, 2, and 5 dpi (one-way ANOVA, 1D *P* < 0.0001, 2D *P* = 0.0002, 3D *P* = 0.8871, and 5D *P* = 0.0002) (Fig. 6A). This contrasts what was observed in *Wolbachia*-free Aag2 cells infected with DENV in which *AeVigilin* was significantly induced in the cells (Fig. 2A). Regarding *AeRACK1*, the transcript levels of the gene were decreased significantly (one-way ANOVA, *P* = 0.0048) only at 1 dpi compared to mock-infected cells (Fig. 6B). However, the transcript levels of the gene increased at 2 and 5 dpi, with no change at 3 dpi compared to mock-infected Aag2.*w*AlbB cells (one-way ANOVA, 2D *P* = 0.0068, 3D *P* = 0.2142, and 5D *P* < 0.0001) (Fig. 6B). Therefore, when comparing *Wolbachia*-free and *Wolbachia*-infected Aag2 cells, DENV infection has the opposite effects on the expression of *AeVigilin*. Furthermore, we did RNAi silencing of these genes in Aag2.*w*AlbB cells to examine the effect on DENV replication. In samples collected at 3 dpi, we first confirmed the silencing of *AeVigilin* and *AeRACK1* (one-way ANOVA, *P* < 0.0001 and *P* = 0.0026, respectively; Fig. 6C and D). There was significant downregulation of DENV gRNA levels in dsVigilin- and dsRACK1-transfected cells compared to Cellfectin-only or dsGFP-transfected controls (one-way ANOVA, *P* < 0.0001 for both genes, respectively; Fig. 6E). These RT-qPCR results were supported by titration assay using supernatants collected from this experiment. DENV virions decreased significantly (one-way ANOVA, *P* = 0.0015 and *P* = 0.03, respectively; Fig. 6F).

We were also interested in looking at *Wolbachia* density in cells with silenced *AeVigilin* and *AeRACK1* genes. We performed qPCR analysis of DNA extracted from the same experiment and found that the density of *Wolbachia* was significantly increased in both RNAi of *AeVigilin* and *AeRACK1* (one-way ANOVA, *P* = 0.0132 and *P* = 0.0008, respectively; Fig. 6G). These results demonstrate that silencing *AeVigilin* and *AeRACK1*, leading to an increase in *Wolbachia* density, might help block the replication of DENV.

### Silencing *AeVigilin* and *AeRACK1* does not affect DENV-2 replication in mosquitoes

**Fig 7:**
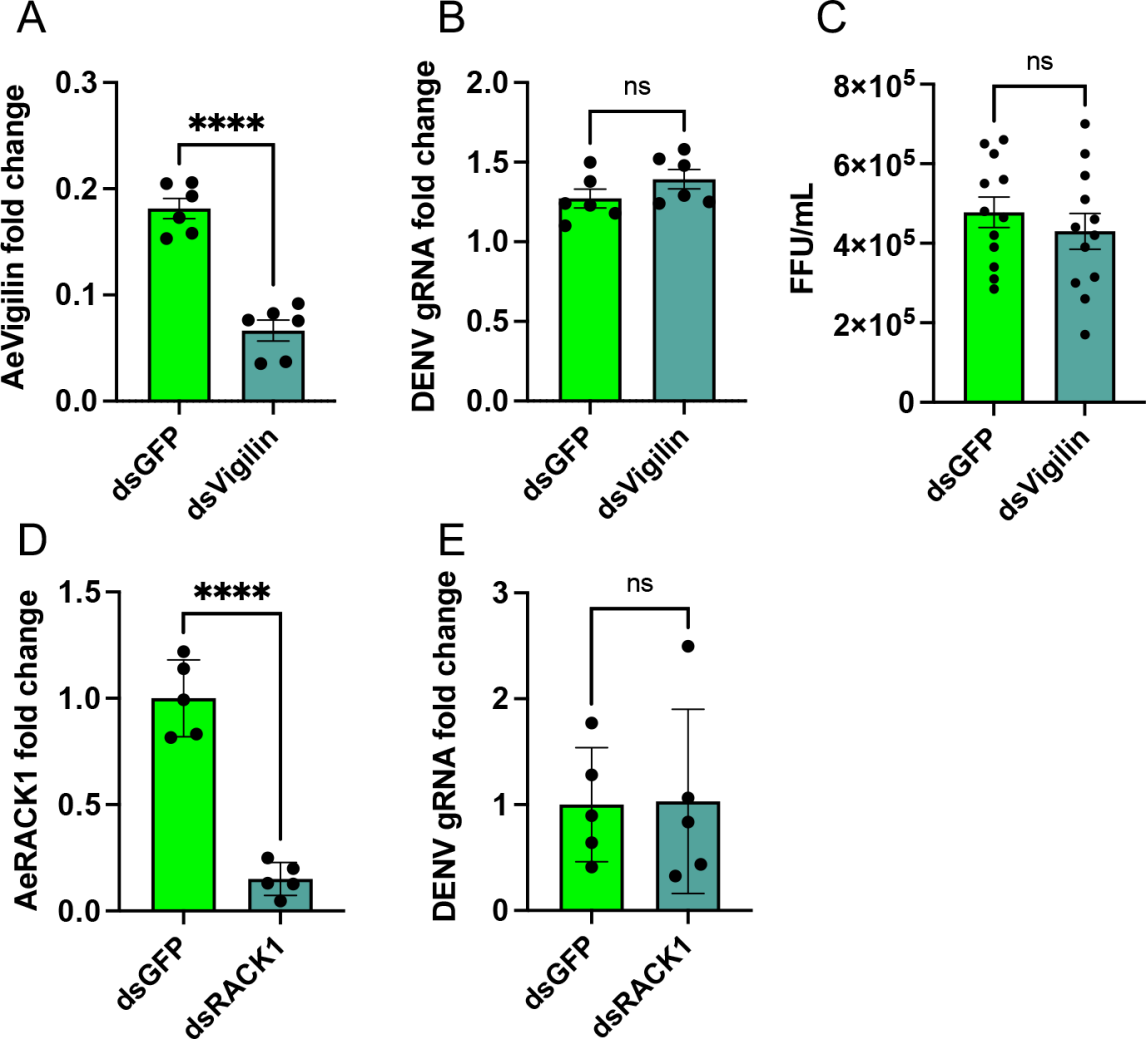
The effect of silencing *AeVigilin* and *AeRACK1* on replication of DENV in *Ae. aegypti* mosquitoes. (**A**) RT-qPCR analysis of RNA extracted from mosquitoes injected with dsVigilin and dsGFP, respectively. (**B**) RT-qPCR analysis of the gRNA levels of DENV in mosquitoes. Mosquitoes were injected with dsVigilin and dsGFP, respectively. (**C**) Focus forming assay to titrate DENV virions in individual mosquitoes injected with dsVigilin and dsGFP. (**D**) RT-qPCR analysis of RNA extracted from mosquitoes injected with dsRACK1 and dsGFP, respectively. (**E**) RT-qPCR analysis of the gRNA levels of DENV in mosquitoes. Mosquitoes were injected with dsRACK1 and dsGFP, respectively. Two days after injection, mosquitoes were fed on DENV and analyzed 4 days following virus feeding. The *t*-test was used to determine statistical differences between groups. The error bars represent the standard error of the mean (SEM) of biological replicates (individual mosquitoes). Asterisks indicate significant differences between compared samples. ****, *P* < 0.0001; ns, not signiﬁcant.

To test whether the effect of silencing *AeVigilin* on DENV in cells is the same as in mosquitoes, 1-day-old female *Wolbachia*-free mosquitoes were injected with dsVigilin and dsGFP, respectively. Two days after injection, mosquitoes were fed with blood containing 1 × 10^7^/mL DENV-2. Mosquitoes that did not feed on blood were discarded, and the remaining mosquitoes were fed with a sugar solution for 4 days. RT-qPCR analysis of RNA extracted from mosquitoes using *AeVigilin* primers showed that this gene was significantly downregulated (63% reduction; *t*-test, *P* < 0.0001) in dsVigilin-injected mosquitoes compared to dsGFP-injected (Fig. 7A). RT-qPCR results showed no significant difference (one-way ANOVA, *P* = 0.1836) in DENV genomic RNA levels in dsVigilin-injected mosquitoes compared to the control group (Fig. 7B). The experiment was repeated three times with similar results. In addition, we quantified DENV virions in individual mosquitoes in a repeat experiment. Similarly, we did not find any difference in DENV titers between dsVigilin and dsGFP-injected mosquitoes (Fig. 7C). Furthermore, when AeRACK1 was silenced in mosquitoes (86% reduction; *t*-test, *P* < 0.0001) (Fig. 7D), we did not find any effect on DENV replication (*t*-test, *P* = 0.9466) (Fig. 7E). These results are different from those in the cell line. This difference could be because cell lines are comprised of homogeneous cells, while the cells and tissues in the mosquito are relatively complex, and simply silencing a single gene may not affect the replication of DENV.

## DISCUSSION

In human cells, the RNA-binding protein Vigilin promotes DENV infection (27), and ribosomal RACK1 interacts with Vigilin to promote DENV replication (35). Both proteins are known to be associated with the ER (35). Here, we investigated the role of *Ae. aegypti* homologs of Vigilin and RACK1 in mosquito-DENV-*Wolbachia* interactions, considering both DENV and *Wolbachia* replicate in association with the ER.

Results showed that both *AeVigilin* and *AeRACK1* were upregulated in *Ae. aegypti* Aag2 cell line when infected with DENV-2. Compared to the controls, the expression levels of *AeRACK1* and *AeVigilin* in DENV-infected mosquitoes were also significantly increased. Therefore, in both DENV-infected mosquitoes and cells the expression of *AeRACK1* and *AeVigilin* were significantly upregulated.

It has been reported that Vigilin mainly exists in the cytosol and is bound to free ribosomes (29), but it has also been reported that a portion of Vigilin is detected in the ER fraction (27). RACK1 is a component of the ribosome 40S subunit located near the mRNA exit channel (30). We separated the cytoplasmic and nuclear fractions of Aag2 and Aag2.*w*AlbB cells. We found that both AeRACK1 and AeVigilin were localized in the cytoplasmic fraction regardless of whether or not cells were infected with DENV. These results confirm that the two proteins are localized in the cytoplasm in both human and mosquito hosts. Furthermore, using co-immunoprecipitation, we showed that AeRACK1 and AeVigilin interact in mosquito cells, consistent with what was observed in human cells.

Studies in human cells using CRISPR-Cas9 knockout identified RACK1 and Vigilin as proviral ER-associated proteins (35). The ability of DENV to infect human HAP1 cells with *RACK1* knockout was significantly reduced (35). Similarly, replication of DENV in *Vigilin*-knocked out HAP1 cells was diminished considerably (35). It appears that RACK1 binds to RNA-binding proteins Vigilin and SERBP1 to facilitate DENV replication in human cells. Both Vigilin and SERBP1 proteins interact with the DENV genome (35). Research also demonstrated that RACK1 interacts with the non-structural protein NS1 from several flaviviruses, including DENV (44). In Aag2 cells, where *AeRACK1* or *AeVigilin* were silenced by RNAi, DENV genomic RNA levels and the number of virions were significantly reduced, although this reduction was more pronounced at the gRNA level (about 50%) compared to virions (within the same log). However, in *AeVigilin* and *AeRACK1*-silenced mosquitoes, there were no significant differences in DENV genomic RNA levels compared with controls. This could be because compared to a cell line, which is rather a homogeneous population of cells, mosquitoes are comprised of different types of cells and tissues, and silencing a gene may not affect DENV replication when analyzed at the whole mosquito level. The studies in human-DENV interactions mentioned above were also done only in cell lines. In summary, our results indicate that *AeRACK1* and *AeVigilin* are important host genes, which promote DENV infection, at least at the cellular level, consistent with studies done in human cell lines.

*Wolbachia* provides anti-viral protection by inhibiting DENV replication in *Ae. aegypti* mosquitoes and cell lines. However, the specific blocking mechanism is unclear. Studies in *Drosophila melanogaster* S2 cells showed that virus blocking occurs in cells in which *Wolbachia* is present, but not in adjacent cells (45). In Aag2.*w*AlbB cells, the expression levels of *AeRACK1* and *AeVigilin* were significantly higher than those in Aag2 cells. In addition, we assessed the expression of *AeRACK1* and *AeVigilin* in mosquitoes transinfected with the *w*AlbB strain of *Wolbachia*. We found that the expression levels of *AeRACK1* and *AeVigilin* were significantly lower than those in mosquitoes without *Wolbachia*. This differs from the results obtained from the cell line, likely due to the complexity of cell types in mosquitoes compared to the homogeneity of cells in the cell line. Furthermore, while most *Wolbachia*-infected Aag2 cells could be infected with *Wolbachia* at similar densities, this may not be the case in mosquitoes. Consequently, this may produce compensatory effects on gene expression in mosquitoes regarding *Vigilin* and *RACK1*, contributing to the differences in their expressions in cell lines and mosquitoes. Considering that *AeRACK1* and *AeVigilin* are proviral genes, the induction of these genes in *Wolbachia*-infected Aag2 cells does not correlate with DENV inhibition in the cells. However, since *Wolbachia*-infected cells block DENV, the induced ER-associated Vigilin and RACK1 proteins may interact with *Wolbachia* proteins, contributing to this inhibition. *Wolbachia* closely interacts with the ER in its host cells, strengthening its relationship for survival and propagation. The ER serves as a membrane source for vacuoles that house *Wolbachia*, facilitating its persistence within cells. This interaction is dynamic, with evidence suggesting that *Wolbachia* modifies the ER membrane composition to suit its needs while avoiding host stress responses such as ER-associated degradation and the unfolded protein response (46). Moreover, *Wolbachia*’s localization near the ER is advantageous for nutrient acquisition, as it utilizes lipids from the host ER to replicate, which can disrupt host lipid metabolism. This strategic positioning also contributes to *Wolbachia*’s ability to block the replication of viruses, as both *Wolbachia* and these viruses compete for similar resources (16, 46, 47). These findings underline the intricate biochemical and structural interplay between *Wolbachia* and the host ER, including its associated proteins. On the other hand, a significant reduction in the expression of *AeRACK1* and *AeVigilin* at the mosquito level may contribute to virus blocking in mosquitoes through a decrease in the amounts of the proviral proteins.

Similar to Aag2 cells, silencing *AeRACK1* and *AeVigilin* in Aag2.*w*AlbB cells led to reductions in DENV replication. However, silencing the genes in Aag2.*w*AlbB cells resulted in significant increases in the density of *Wolbachia*. Therefore, silencing *AeRACK1* and *AeVigilin*, which are proviral, and increasing the density of *Wolbachia* may jointly reduce the replication of DENV genomic RNA and the number of DENV virions in Aag2.*w*AlbB cells.

In summary, our results show that DENV induces the expression of *AeRACK1* and *AeVigilin* at the gene and protein levels in Aag2 cells, and also upregulates the expression of *AeRACK1* and *AeVigilin* in *Ae. aegypti* mosquitoes. The two proteins are mainly localized to the cytoplasm, and they interact with each other. Silencing *AeRACK1* and *AeVigilin* in Aag2 cells significantly reduced the replication of DENV genomic RNA and the number of DENV virions; however, silencing *AeVigilin* or *AeRACK1* did not affect DENV genomic RNA levels in *Ae. aegypti* mosquitoes. In addition, we found that in the presence of *Wolbachia AeRACK1* and *AeVigilin* are upregulated in cells but downregulated in *Ae. aegypti* mosquitoes. Downregulation of the genes in mosquitoes, which are proviral, may contribute to the virus-blocking mechanism. However, further investigations are required to understand the role of these genes in the blocking mechanism. For example, we did not explore the genes in association with other *Wolbachia* strains or in different hosts. The other aspect that could have been explored is whether *Wolbachia* could make AeVigilin and AeRACK1 less accessible in the ER, considering it is the main site of DENV replication. Overall, our results suggest that AeRACK1 and AeVigilin are involved in DENV replication in mosquitoes and may contribute to virus blocking by *Wolbachia*.
